# Titanium peroxide nanoparticles enhanced cytotoxic effects of X-ray irradiation against pancreatic cancer model through reactive oxygen species generation *in vitro* and *in vivo*

**DOI:** 10.1186/s13014-016-0666-y

**Published:** 2016-07-07

**Authors:** Masao Nakayama, Ryohei Sasaki, Chiaki Ogino, Tsutomu Tanaka, Kenta Morita, Mitsuo Umetsu, Satoshi Ohara, Zhenquan Tan, Yuya Nishimura, Hiroaki Akasaka, Kazuyoshi Sato, Chiya Numako, Seiichi Takami, Akihiko Kondo

**Affiliations:** Division of Radiation Oncology, Kobe University Graduate School of Medicine, 7-5-2 Kusunokicho, Chuouku, Kobe, Hyogo 650-0017 Japan; Department of Chemical Science and Engineering, Graduate School of Engineering, Kobe University, 1-1 Rokkoudaicho, Nadaku, Kobe, Hyogo 657-8501 Japan; Department of Biomolecular Engineering, Graduate School of Engineering, Tohoku University, 6-6 Aramaki, Aza, Aobaku, Sendai, Miyagi 980-8579 Japan; Joining and Welding Research Institute, Osaka University, 11-1 Mihogaoka, Ibaraki, Osaka 567-0047 Japan; Division of Environmental Engineering Science, Graduate School of Science and Technology, Gunma University, 1-5-1 Tenjincho, Kiryu, Gunma 376-8515 Japan; Graduate School of Science, Chiba University, 1-33 Yayoi, Inage, Chiba, 263-8522 Japan; Institute of Multidisciplinary Research for Advanced Materials, Tohoku University, 2-1-1 Katahira, Aobaku, Sendai, Miyagi 980-8577 Japan

**Keywords:** Nanoparticle, Titanium peroxide, Radiation, Reactive oxygen species, Pancreatic cancer

## Abstract

**Background:**

Biological applications of nanoparticles are rapidly increasing, which introduces new possibilities to improve the efficacy of radiotherapy. Here, we synthesized titanium peroxide nanoparticles (TiOxNPs) and investigated their efficacy as novel agents that can potently enhance the effects of radiation in the treatment of pancreatic cancer.

**Methods:**

TiOxNPs and polyacrylic acid-modified TiOxNPs (PAA-TiOxNPs) were synthesized from anatase-type titanium dioxide nanoparticles (TiO_2_NPs). The size and morphology of the PAA-TiOxNPs was evaluated using transmission electron microscopy and dynamic light scattering. The crystalline structures of the TiO_2_NPs and PAA-TiOxNPs with and without X-ray irradiation were analyzed using X-ray absorption. The ability of TiOxNPs and PAA-TiOxNPs to produce reactive oxygen species in response to X-ray irradiation was evaluated in a cell-free system and confirmed by flow cytometric analysis in vitro. DNA damage after X-ray exposure with or without PAA-TiOxNPs was assessed by immunohistochemical analysis of γ-H2AX foci formation in vitro and in vivo. Cytotoxicity was evaluated by a colony forming assay in vitro. Xenografts were prepared using human pancreatic cancer MIAPaCa-2 cells and used to evaluate the inhibition of tumor growth caused by X-ray exposure, PAA-TiOxNPs, and the combination of the two.

**Results:**

The core structures of the PAA-TiOxNPs were found to be of the anatase type. The TiOxNPs and PAA-TiOxNPs showed a distinct ability to produce hydroxyl radicals in response to X-ray irradiation in a dose- and concentration-dependent manner, whereas the TiO_2_NPs did not. At the highest concentration of TiOxNPs, the amount of hydroxyl radicals increased by >8.5-fold following treatment with 30 Gy of radiation. The absorption of PAA-TiOxNPs enhanced DNA damage and resulted in higher cytotoxicity in response to X-ray irradiation in vitro. The combination of the PAA-TiOxNPs and X-ray irradiation induced significantly stronger tumor growth inhibition compared to treatment with either PAA-TiOxNPs or X-ray alone (*p* < 0.05). No apparent toxicity or weight loss was observed for 43 days after irradiation.

**Conclusions:**

TiOxNPs are potential agents for enhancing the effects of radiation on pancreatic cancer and act via hydroxyl radical production; owing to this ability, they can be used for pancreatic cancer therapy in the future.

**Electronic supplementary material:**

The online version of this article (doi:10.1186/s13014-016-0666-y) contains supplementary material, which is available to authorized users.

## Background

Pancreatic cancer is a highly lethal disease that is often diagnosed only in the advanced stage. It is the fourth most common cause of cancer-related deaths in the United States, causing 40,560 deaths annually [1]. Moreover, the 5-year survival rate of pancreatic cancer patients is approximately 3-7 % after diagnosis [1, 2]. Locally advanced pancreatic cancer is also notoriously resistant to many types of cytotoxic chemotherapy and radiotherapy [3]. As a result, there are currently no effective therapies for pancreatic cancer, and novel strategies need to be explored.

The biological application of nanoparticles (NPs) is rapidly increasing in nanotechnology and introduces new possibilities for the diagnosis and treatment of human cancers [4–6]. NPs have been used in many different areas of radiation oncology, including radiosensitization [7]. Nano-sized titanium dioxide (TiO_2_) is one of the most widely produced nanoparticles. TiO_2_ is poorly soluble and has been used in numerous applications as a food colorant, or as a white pigment in a number of products including cosmetics, medicines, and pharmaceutical products [8, 9]. Rutile and anatase are the two major crystalline forms of TiO_2_. The photocatalytic activity and cytotoxicity of anatase TiO_2_ nanoparticles (TiO_2_NPs) are higher than those of the rutile forms [10, 11]. Recently, TiO_2_NPs have been used in the phototherapy of malignant cells and are regarded as potential photosensitizing agents for photodynamic therapy because they exert unique phototoxic effects upon ultraviolet (UV) irradiation [12–16]. Despite the promising effects of UV-activated TiO_2_NPs, this strategy seems to be ineffective in treating many cancers and is difficult to apply clinically for two major reasons. First, UV light cannot penetrate the human body to reach internal organs such as the gastrointestinal system, liver, and pancreas, thus limiting the application of this technique to superficial tumors [17]. Second, the UV-mediated production of reactive oxygen species (ROS) occurs for a very short duration and is insufficient to provide a continuous and prolonged cancer-killing effect [18]. Radiotherapy using X-rays is known to be effective in various cancer treatments. However, to our knowledge, there are few reports investigating whether or not TiO_2_NPs can enhance the effects of X-ray irradiation [19]. In addition, the properties of TiO_2_NPs seem to be insufficient to render them useful as radiosensitizers. Wang et al. demonstrated that the potential biological effects of TiO_2_NPs depend on their size, crystal phase, surface coating, and chemical composition [20]. Therefore, certain modifications may be necessary to make TiO_2_NPs suitable for use as agents that enhance the effects of radiation.

Chemical reactions between hydrogen peroxide and TiO_2_ have been widely investigated [21, 22], but the effects of these reactions on the properties of TiO_2_NPs in response to X-ray irradiation have not been clarified. In this study, we investigated the properties of titanium peroxide nanoparticles (TiOxNPs) to determine whether the TiOxNPs might be useful as potential agents to enhance the effects of radiation against a human pancreatic cancer model in vitro and in vivo.

## Materials and methods

### Preparation of TiOxNPs

**Fig. 1:**
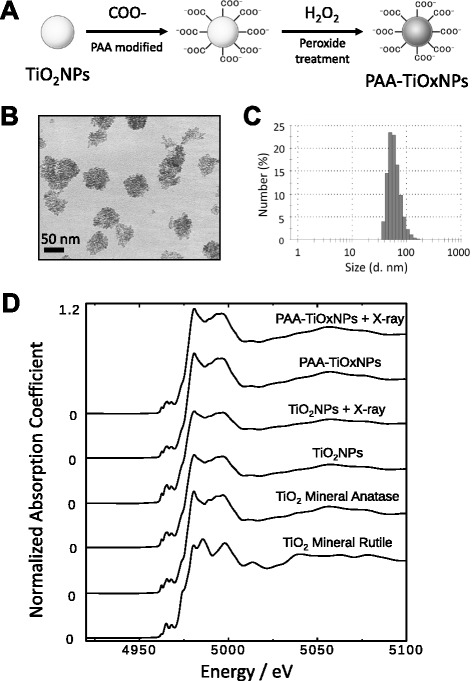
Characteristics of the PAA-TiOxNPs. **a** Scheme for the synthesis of the PAA-TiOxNPs from anatase TiO_2_NPs. **b** Representative TEM image of the PAA-TiOxNPs. The size of the PAA-TiOxNPs was approximately 50 nm. **c** Size distribution of the PAA-TiOxNPs measured by DLS. **d** Structure of TiO_2_NPs and PAA-TiOxNPs before and after X-ray irradiation, as determined by XAFS spectra

TiOxNPs were synthesized from anatase-type TiO_2_NPs according to several procedures that involve hydrogen peroxide processing (Fig. 1a) [23]. For in vitro and in vivo experiments, the surfaces were modified using polyacrylic acid (PAA) to prevent aggregation of the bare TiOxNPs under physiological conditions [24]. The material for the TiO_2_NPs (STS-01) was purchased from Ishihara Sangyo, Ltd. (Osaka, Japan). Details of the syntheses of TiOxNPs and PAA-TiOxNPs are shown in Additional file 1.

### Transmission electron micrography (TEM), dynamic light scattering (DLS), and X-ray absorption fine structure (XAFS) analyses

The size and morphology of the PAA-TiOxNPs were studied using a transmission electron microscope (JEM-1200EX, JEOL Ltd., Tokyo, Japan) as described previously [25]. The transmission electron micrographs were recorded at an acceleration voltage of 80 kV. DLS was conducted using a Malvern Zetasizer Nano ZS to estimate the hydrodynamic diameter of the PAA-TiOxNPs.

The XAFS measurements in fluorescence mode were performed around a Ti K-edge energy region at BL9A at the Photon Factory, KEK, Japan. Synchrotron radiation from a 2.5 GeV storage ring was monochromatized using a Si (111) double crystal monochromator modified to 2 × 1 mm^2^ with slits and counted using an ion chamber for I0 and a Lytle-type detector for fluorescence X-rays. Higher harmonic radiation was removed using an Rh-Ni-coated mirror. The TiO_2_NP and PAA-TiOxNP suspensions were put into polyethylene bags for the XAFS measurements. TiO_2_ minerals (rutile and anatase) on Scotch tape were measured as standard titanium materials with known chemical compositions and crystal structures.

### Cell culture and establishment of the animal models

The MIAPaCa-2 human pancreatic cancer cell line was obtained from the American Type Culture Collection (Rockville, MD, USA) and maintained in RPMI-1640 medium. Male BALB/c nude mice (body weight: 20–22 g) were purchased from CLEA Corporation (Tokyo, Japan). The nude mice were maintained in specific pathogen-free animal care facilities under isothermal conditions with regular photoperiods. All animal experiments were approved by the Institutional Animal Care and Use Committee (Permission number: 100605R1) and performed according to Kobe University Animal Experimentation Regulations.

### X-ray irradiation

X-ray irradiation was performed using a MBR-1505R2 (Hitachi, Tokyo, Japan) at a voltage of 150 kV and a current of 5 mA with a 1-mm-thick aluminum filter (0.5 Gy/min at the target). Prior to each experiment, the mice were anesthetized using an intraperitoneal administration of somnopentyl (0.1 mg/g body weight) and were then put to sleep under anesthesia and immobilized in a customized harness that exposed the hind leg while shielding the remainder of the body with lead during irradiation.

### ROS evaluation

Quantification of ROS generation from the NPs in response to X-ray irradiation in a cell-free system was performed using 3’-(p-aminophenyl) fluorescein (APF; Sekisui Medical Co. Ltd., Tokyo, Japan), which fluoresces mainly in response to hydroxyl radicals [26]. APF (5 μM) was added to different concentrations of the suspensions of TiOxNPs, PAA-TiOxNPs, and TiO_2_NPs prepared using 96-well plates. Each plate was then exposed to different doses of X-ray radiation. The APF signal was measured using a multi-well plate reader (Fluoroskan Ascent FL, Thermo Fisher Scientific Inc., MA, USA) at excitation/emission wavelengths of 490/515 nm. To determine whether the increase in APF fluorescent signals was caused by ROS generation, antioxidants such as vitamin C (1 mM) or glutathione (1 mM) were added as ROS scavengers. The amount of hydrogen peroxide was measured in response to carboxy-2′, 7’-dichlorofluorescein (C-H_2_DCF; Molecular Probes, Inc., Eugene, OR, USA). C-H_2_DCF (50 μM) was added to suspensions of TiOxNPs, which were then exposed to X-ray radiation. The fluorescent signal was measured at excitation/emission wavelengths of 485/612 nm.

The production of intracellular ROS including hydrogen peroxide, hydroxyl radicals, and superoxide anions was evaluated using a fluorescence activated cell sorting (FACS Calibur; Becton-Dickinson, NJ, USA). The MIAPaCa-2 cells were treated with 0.1 % w/v PAA-TiOxNPs for 1 h at 37 °C with or without 30 Gy X-ray irradiation. For 30 min, the cells were stained with 10 μM APF, 50 μM C-H_2_DCF, and 50 ng/mL hydroethidium (HE; Molecular Probes, Inc., OR, USA) per sample to detect cellular hydroxyl radicals, hydrogen peroxide, and superoxide anions, respectively [27].

### Detection of DNA damage and colony forming assay in vitro

Induction of deoxyribonucleic acid (DNA) damage was investigated by the detection of phosphorylated histone 2AX (γ-H2AX) foci using immunocytochemistry. MIAPaCa-2 cells were subcultured on 35-mm dishes. The cells were treated with 0.15 % w/v PAA-TiOxNPs for 1 h and/or 5 Gy of X-ray irradiation. After the treatment, the cells were fixed in 4 % paraformaldehyde in phosphate buffered saline (PBS) for 20 min, permeabilized with 0.1 % Triton X-100 in PBS for 5 min, and blocked in 5 % bovine serum albumin in PBS for 60 min. Cells were incubated with 1:200 rabbit anti γ-H2AX antibody (Cell Signaling Technology, MA, US) overnight at 4 °C. Then, the cells were incubated with 1:20 tetramethyl rhodamine isothiocyanate (TRITC)-conjugated anti-rabbit secondary antibody (Dako, Glostrup, Denmark) for 90 min at room temperature. Cell nuclei were stained with 4’, 6-diaidino-2-phenylindole (DAPI). Stained cells were observed using a fluorescence microscope (BZ-9000, KEYENCE, Osaka, Japan). Cells expressing nuclear γ-H2AX foci were then counted manually from 100 cells for each treatment, and the data were presented as the mean ± standard deviation (SD) from 3 fields for each section [28].

To evaluate whether the PAA-TiOxNPs might enhance the effects of radiation in vitro, a colony forming assay was performed. MIAPaCa-2 cells (1 × 10^6^) were exposed to 0.15 % w/v PAA-TiOxNPs for 30 min, and then they were treated with 0, 3, or 5 Gy of X-ray irradiation. The treated cells were counted and replated onto a new 10-cm tissue culture dish with fresh medium not containing PAA-TiOxNPs. The cells were incubated for 10 days until the cell population completed colony formation. After fixing and staining, colonies consisting of more than 50 cells were counted and the surviving fractions were calculated based on the survival of non-irradiated cells.

### Tumor growth inhibition of PAA-TiOxNPs combined with X-ray radiation

MIAPaCa-2 cells (2 × 10^6^) were injected subcutaneously into the hind legs of the BALB/c nude mice as described previously [29]. The tumor was expected to enlarge to be palpable, i.e., approximately 6–10 mm in the longest axis and 5–7 mm in the shortest axis, with skin thickness by 7 days post injection. Tumor volume was calculated using the formula L × W^2^/2, where L is the longest axis and W is the shortest axis of the tumor. Using this formula, the tumor volume was approximately 100–200 mm^3^ on the treatment day. The mice were stratified into 4 subgroups consisting of 3 mice each: untreated, PAA-TiOxNPs alone, X-rays alone, and X-rays combined with PAA-TiOxNPs. The mice were given an intra-tumoral injection of 150 μL of an 8.7 % w/v PAA-TiOxNP suspension with or without a single dose of 5 Gy of X-ray irradiation approximately 1 h after the injection. To inject a sufficient amount of the PAA-TiOxNP suspension while keeping the suspension as homogeneous as possible, we injected 150 μL of the PAA-TiOxNP suspension into each tumor. The tumor size, body weight, and health of the mice were measured for 43 days after the initial treatment (for a total of 50 days after the injection) [30].

### Mechanism of tumor growth inhibitory effect by PAA-TiOxNPs combined with X-ray irradiation

At 24 h after treatment, the tumors were excised, fixed in 10 % formalin, and embedded in paraffin sections (4-μm-thick). The samples were stained using hematoxylin and eosin (H-E).

Induction and maintenance of DNA damage in the tumor by X-ray radiation with or without PAA-TiOxNPs was evaluated by detection of γ-H2AX immunoreactivity using a fluorescence microscope (BZ-9000, Keyence, Osaka, Japan). Sections were counterstained with DAPI. Positive γ-H2AX signals in the nucleus were visually counted in 300 cells for each treatment. The number of γ-H2AX foci was calculated by averaging the number of the positive cells from 3 fields for each section. Induction of apoptosis was also evaluated using the terminal deoxynucleotidyl transferase-mediated deoxyuridine triphosphate nick end labeling (TUNEL) assay (Roche Applied Science, Indianapolis, IN, US) [31]. Positive TUNEL signals were evaluated in 100 cells for each treatment, and the number of apoptotic cells was calculated by averaging the number of positive TUNEL signals from 6 fields for each section.

### Statistical analysis

Data are expressed as the mean ± SD. Comparisons were performed using Student’s *t*-test, and differences were considered significant at the 95 % confidence level (*p* < 0.05).

## Results

### Successful preparation of TiOxNPs

Considering the enhanced permeability and retention effect, we aimed to prepare nanoparticles with a size of less than 100 nm. The PAA-TiOxNPs were found to be approximately 50–70 nm in diameter, as determined using TEM (Fig. 1b). Consistent with the TEM images, the size of the PAA-TiOxNPs, as determined using DLS, was approximately 50–100 nm with a narrow unimodal size distribution (Fig. 1c).

The crystalline structures of TiO_2_NPs and PAA-TiOxNPs with or without X-ray radiation (16 Gy) were analyzed using XAFS (Fig. 1d). No change in the coordination environment was observed around the Ti ion from the original anatase-type TiO_2_NPs until the PAA-TiOxNPs were irradiated with X-rays. From this observation, it was concluded that the PAA-TiOxNPs preserve the anatase structure of TiO_2_NPs in the core structures.

### ROS-generating effect of TiOxNPs under X-ray irradiation

**Fig. 2:**
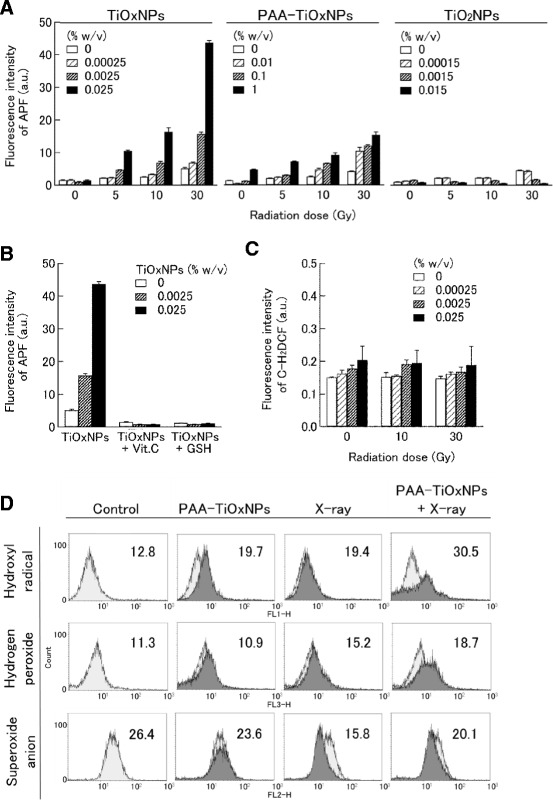
ROS production by the TiOxNPs, PAA-TiOxNPs, and TiO_2_NPs under X-ray irradiation. **a** APF intensity indicating that hydroxyl radical production in the TiOxNPs and the PAA-TiOxNPs increased in a radiation dose-dependent manner, but that of the TiO_2_NPs did not. Irradiated radiation doses were 0, 5, 10, and 30 Gy. Data are shown as the mean ± SD from 5 independent experiments. **b** Production and scavenging of ROS by 1 mM vitamin C (Vit. C) or 1 mM glutathione (GSH). Histograms show mean ± SD calculated from 5 independent experiments. **c** Hydrogen peroxide production from the TiOxNPs under X-ray irradiation. **d** Detection of intracellular ROS production by FACS. Mean fluorescence values are shown in each figure

The amount of hydroxyl radicals increased in a radiation dose- and nanoparticle concentration-dependent manner in the TiOxNPs and PAA-TiOxNPs, whereas no increase in hydroxyl radical levels was observed in the TiO_2_NPs (Fig. 2a). At the highest concentration of TiOxNPs and PAA-TiOxNPs, the amount of hydroxyl radicals with 30 Gy of radiation increased by more than 8.5 and 3.7 fold, respectively. The ROS-generating effect using bare TiOxNPs appeared to be stronger than that of PAA-TiOxNPs. ROS production was also confirmed using antioxidants such as vitamin C and glutathione. When these antioxidants were added to the TiOxNP suspension, the APF signal did not increase, even with a radiation dose of 30 Gy (Fig. 2b). Thus, the TiOxNPs could produce large amounts of hydroxyl radicals under X-ray irradiation. On the contrary, hydrogen peroxide production from the TiOxNPs did not increase under X-ray irradiation (Fig. 2c).

To assess intracellular ROS production by PAA-TiOxNPs with X-ray radiation, the amount of hydroxyl radicals, hydrogen peroxide, and superoxide anions was measured using FACS. In cells treated with PAA-TiOxNPs combined with X-ray radiation, the hydroxyl radical levels increased by more than 2 fold and the hydrogen peroxide levels increased by more than 1.6 fold in comparison to the control, whereas the superoxide levels did not increase (Fig. 2d).

### Cytotoxic effects of PAA-TiOxNPs under X-ray irradiation in vitro

**Fig. 3:**
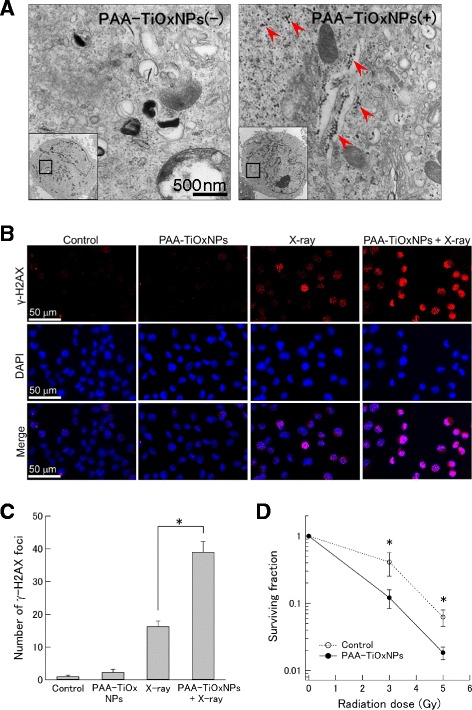
PAA-TiOxNPs enhanced the effects of radiation *in vitro*. **a** TEM images showing PAA-TiOxNPs within the MIAPaCa-2 cells (red arrows). **b**, **c** DNA damage illustrated by immunohistochemistry of γ-H2AX foci in the nucleus. The X-ray irradiation combined with PAA-TiOxNPs induced a higher proportion of γ-H2AX foci-positive cells compared to the single treatment of X-ray irradiation or PAA-TiOxNPs. Data are shown as the mean ± SD. *p < 0.05. **d** Significantly higher combination effects of X-ray irradiation with PAA-TiOxNPs were observed in a colony forming assay. Data are shown as the mean ± SD from 3 independent experiments. *p < 0.05

First, intracellular absorption of PAA-TiOxNPs in MIAPaCa-2 cells was directly confirmed by TEM (Fig. 3a). Next, DNA damage induced by X-ray irradiation with or without PAA-TiOxNPs was quantified with regard to γ-H2AX foci formation. Compared to X-ray irradiation alone, combination with PAA-TiOxNPs induced a higher proportion of γ-H2AX foci (*p* < 0.05, Fig. 3b, c). The effect of the combination of PAA-TiOxNPs and X-ray irradiation evaluated by a colony forming assay was significantly better than that of X-rays alone (*p* < 0.05, Fig. 3d). Overall, the PAA-TiOxNPs enhanced the cytotoxic effect of X-ray irradiation in vitro.

### Tumor growth inhibitory effects of PAA-TiOxNPs combined with X-ray irradiation in vivo

**Fig. 4:**
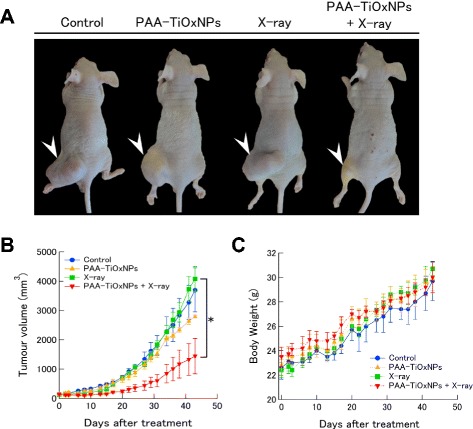
Tumor growth-inhibitory effects of PAA-TiOxNPs combined with X-ray radiation. **a** Tumor appearance in the xenografts for each treatment after 43 days (arrowhead). **b** Changes in tumor size after each treatment. Each group consisted of 3 mice. Data are shown as the mean ± SD. *p < 0.05. **c** Body weight changes after each treatment

The tumor growth inhibition in the group receiving both the PAA-TiOxNPs and X-ray treatments was significantly greater than that in the groups that received a single treatment or in the untreated subgroups at 43 days after the initial treatment (*p* < 0.05, Fig. 4a, b). The tumor volume in the mice treated with PAA-TiOxNPs and X-rays was 35.4 % of that in mice treated with X-rays alone. Thus, the PAA-TiOxNPs in combination with X-ray radiation had synergistic cytotoxic effects in vivo.

No immediate toxic reaction was observed in the mice injected with the PAA-TiOxNPs. No mice died during the 43-day observation period and none showed any apparent loss of body weight (Fig. 4c). These findings suggest that the PAA-TiOxNPs themselves do not have any apparent toxicity at the effective dose applied during this period.

### Mechanisms underlying tumor growth inhibitory effects in vivo

**Fig. 5:**
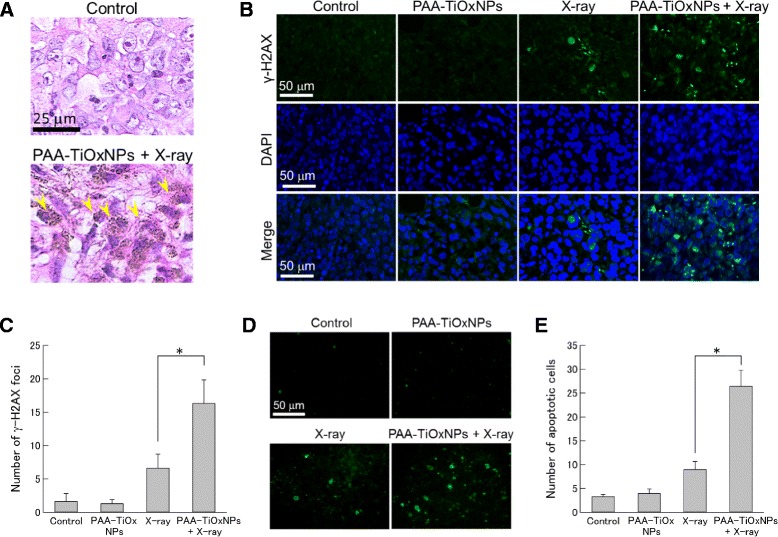
Detection of DNA damage and of apoptosis induced by X-ray irradiation, the PAA-TiOxNPs, and their combination *in vivo*. **a** Localization of the PAA-TiOxNPs (as visualized using H-E staining). Brown dots indicate accumulation of the PAA-TiOxNPs (yellow arrows). **b** Detection of DNA damage by staining of γ-H2AX and DAPI in MIAPaCa-2 cells. **c** The number of γ-H2AX foci with the combination treatment was significantly higher than that with the single treatment. Data are shown as the mean ± SD. *p < 0.05. **d** Induction of apoptosis and its evaluation by TUNEL assay. **e** The number of apoptotic cells with the combination treatment was significantly higher than that with the single treatment. Data are shown as the mean ± SD. *p < 0.05

The localization of the injected PAA-TiOxNPs was evaluated histologically. In H-E stained sections, PAA-TiOxNPs were observed as brown dots (Fig. 5a). The brown dots indicating aggregated PAA-TiOxNPs were observed inside the tumor cells. Immunoreactivity of γ-H2AX indicating occurrence and maintenance of DNA damage in the tumor specimen was evaluated. The PAA-TiOxNPs combined with X-ray irradiation induced significantly higher numbers of positive cells compared to PAA-TiOxNPs or X-ray irradiation alone (*p* < 0.05, Fig. 5b, c). The number of TUNEL-positive cells was also significantly higher in the combination group than in the single treatment or untreated subgroups (*p* < 0.05, Fig. 5d, e). These findings indicate that the PAA-TiOxNPs enhanced the cytotoxicity of X-ray radiation through DNA damage and induction of apoptosis.

## Discussion

To our knowledge, this is the first report to show that TiOxNPs synthesized from anatase-type TiO_2_NPs can produce hydroxyl radicals under X-ray irradiation. This property resulted in remarkable tumor growth inhibition in a human pancreatic carcinoma xenograft.

The core of the PAA-TiOxNPs remained the same as that of TiO_2_NPs with the original anatase structure (Fig. 1d), whereas the property of the TiOxNPs to produce ROS in response to X-ray irradiation was different from that of TiO_2_NPs (Fig. 2a). Based on these findings and according to the process used to synthesize the TiOxNPs, it was speculated that surface of the TiOxNPs was different from that of TiO_2_NPs, and it seemed to be peroxidized by hydrogen peroxide. The acquired property of the TiOxNPs was first examined under cell-free conditions (only water and TiOxNPs) using APF. Because the APF reaction is specific to hydroxyl radicals, peroxynitrite anion, and hypochlorite anion [26], the major ROS generated from the TiOxNPs and PAA-TiOxNPs in response to X-ray irradiation was identified as hydroxyl radicals. Next, this finding was further investigated in cells using FACS (Fig. 2d). The results indicated that the hydroxyl radical and hydrogen peroxide levels increased, whereas the levels of superoxide anions did not. These results were consistent with those observed in the cell-free system. Cellular redox homeostasis is maintained by the balance between the generation and elimination of ROS. Exogenous agents that increase ROS generation or decrease antioxidant capacity will shift the redox balance and result in an overall increase in the ROS levels, which may induce cell death when above a cellular tolerability threshold [32]. Cancer cells would be more dependent on the antioxidant system and more vulnerable to further oxidative stress induced by exogenous ROS-generating agents or compounds that inhibit the antioxidant system. Combinations of ROS-generating agents with X-ray radiation, which is capable of abrogating cellular antioxidant systems, are likely to have an additive or synergistic cytotoxic effect. From our determination that the combination of X-ray irradiation and PAA-TiOxNPs induced higher amounts of ROS and DNA damage in vitro, it was at least speculated that the PAA-TiOxNPs might act as ROS-generating agents in response to X-ray irradiation in the cells. The mechanism might affect the combination effect observed in the colony forming assay (Fig. 3d).

It was noteworthy that the use of PAA-TiOxNPs in combination with X-ray radiation induced tumor growth inhibition in vivo. The effect of the combination therapy was significantly greater than that of each of the single treatments (Fig. 4a, b), thus indicating a potential synergistic effect. However, although our results indicate a potential role for the production of hydroxyl radicals from the PAA-TiOxNPs, the sites for ROS production (inside the cells, or on the outer membrane, or both) remain unclear. In the histochemical analyses, the PAA-TiOxNPs appeared to accumulate inside the cells (Fig. 5a), thus suggesting that the increase in ROS levels according to the Fenton reaction may occur inside the cells. Halliwell et al. previously reported that the hydroxyl radical could be produced via the Fenton reaction in the presence of biological free iron and superoxide, leading to oxidative damage [33]. In our experimental setting, a small radiation dose (5 Gy) was effective for induction of apoptosis and tumor shrinkage; larger doses or multiple doses will probably have greater effects. The use of the combination of PAA-TiOxNPs with X-ray radiation in vivo appeared to be effective for inhibiting tumor growth.

Metal nanoparticles have unique characteristics such as particle size, high surface-to-volume ratio, broad optical properties, and facile surface chemistry [34, 35]. Radiosensitization of gold nanoparticles (GNPs) occurs because of the high absorbance of gold and the resulting deposition of energy in surrounding tissues from photoelectrons and auger electrons, and because of the generation of ROS [36–39]. Compared to these findings for GNPs, fewer data appear to be available for the applications of TiOxNPs, and multiple aspects of TiOxNPs still need to be evaluated. Although our results indicate that TiOxNPs have potential for use as agents that enhance the effects of radiation, further research on this property of TiOxNPs in comparison to GNPs is necessary.

Our study has a few limitations. The route of administration was intra-tumoral injection, and the effects of intravenous injection were not tested. There are several reports indicating that intravenously injected GNPs can readily extravasate into advanced brain tumors, leading to increased survival of mice with advanced glioblastoma treated with radiotherapy plus GNPs [40, 41]. Therefore, systemic intravenous administration of PAA-TiOxNPs may induce an additional effect. Further evaluation is warranted in this regard. On the contrary, some studies have reported protracted elimination of GNPs from the liver [42, 43], and other studies have reported the nephrotoxicity of GNPs [44, 45]. Although no change in the weight of the mice was observed in our experimental setting, larger amounts of PAA-TiOxNPs seem to be necessary in case of systemic administration, and toxicity, including acute liver or renal toxicity, will need to be carefully evaluated.

## Conclusions

In conclusion, TiOxNPs showed remarkable ROS production upon X-ray irradiation. PAA-TiOxNPs were effective in a mouse model using engrafted human pancreatic cancer cells. Although future studies will be required to confirm the therapeutic effects and potential toxicity of nanoparticles, our study shows that TiOxNPs are promising agents for enhancing the effects of radiation in pancreatic cancer therapy.

## Abbreviations

APF, 3’-(p-aminophenyl) fluorescein; C-H_2_DCF, carboxy-2′, 7’-dichlorofluorescein; DAPI, 4′, 6-diaidino-2-phenylindole; DLS, dynamic light scattering; DNA, deoxyribonucleic acid; FACS, fluorescence activated cell sorting; GNPs, gold nanoparticles; H-E, hematoxylin and eosin; HE, hydroethidium; NPs, nanoparticles; PAA, polyacrylic acid; PBS, phosphate buffered saline; ROS, reactive oxygen species; SD, standard deviation; TEM, transmission electron micrography; TiO_2_, titanium dioxide; TiO_2_NPs, titanium dioxide nanoparticles; TiOxNPs, titanium peroxide nanoparticles; TUNEL, terminal deoxynucleotidyl transferase-mediated deoxyuridine triphosphate nick end labeling; UV, ultraviolet; XAFS, X-ray absorption fine structure; γ-H2AX, phosphorylated histone 2AX

## Additional file

Additional file 1: Details of the synthetic protocol for PAA-TiOxNPs formation from TiO_2_NPs. (PPTX 151 kb)
