# Supplementary material for: Titanium peroxide nanoparticles enhanced cytotoxic effects of X-ray irradiation against pancreatic cancer model through reactive oxygen species generation in vitro and in vivo
Source: Radiat Oncol. 2016 Jul 7;11:91. doi: 10.1186/s13014-016-0666-y (PMC4936232; doi:10.1186/s13014-016-0666-y)
Supplement: Additional file 1: — Details of the synthetic protocol for PAA-TiOxNPs formation from TiO2NPs. (PPTX 151 kb) [file 13014_2016_666_MOESM1_ESM.pptx]

## Slide 1
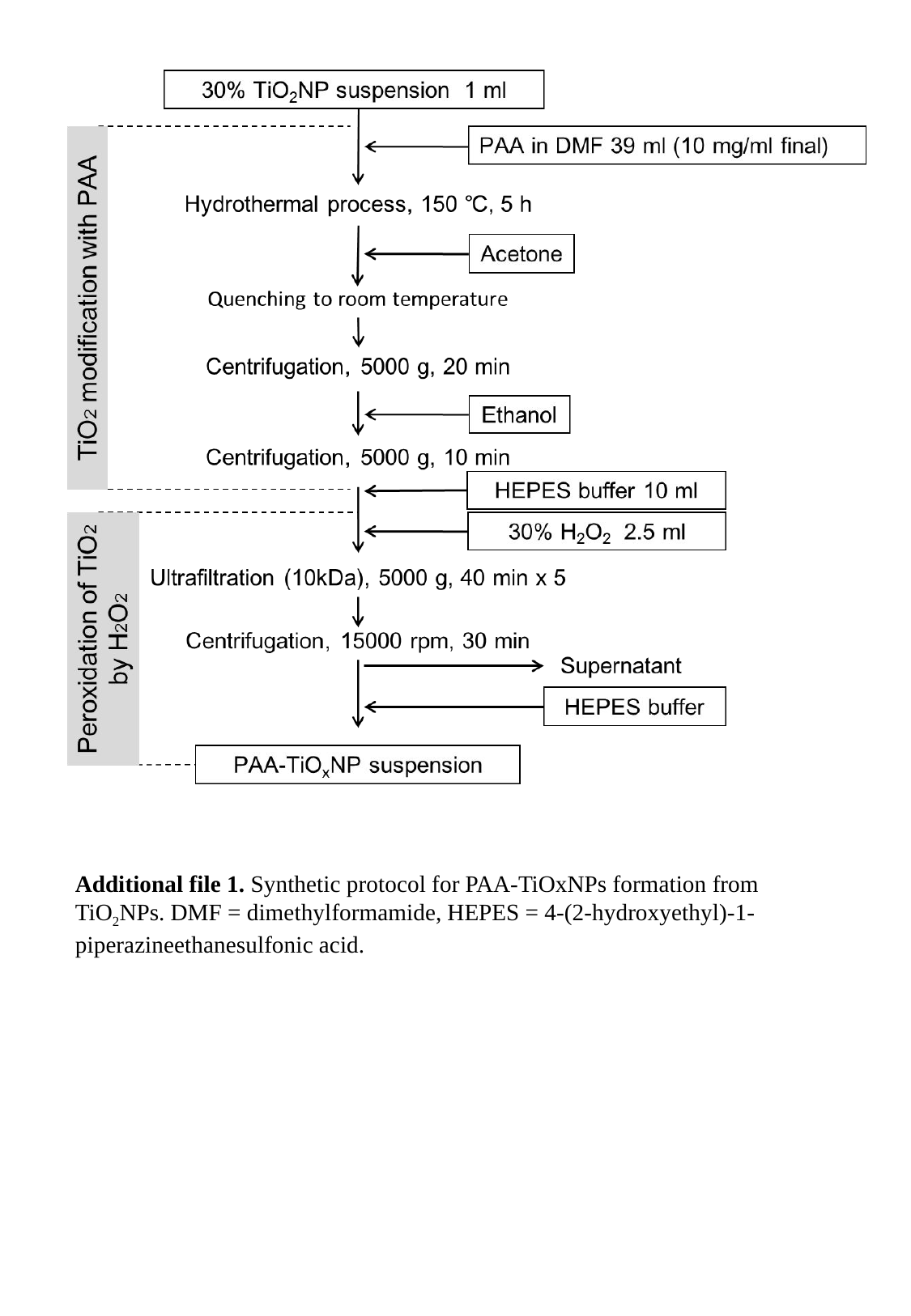

Additional file 1. Synthetic protocol for PAA-TiOxNPs formation from TiO2NPs. DMF = dimethylformamide, HEPES = 4-(2-hydroxyethyl)-1-piperazineethanesulfonic acid.
